# Do we have enough evidences that make you safe to treat a man with hypogonadism one year after a radical prostatectomy for prostate cancer? | *Opinion: YES*

**DOI:** 10.1590/S1677-5538.IBJU.2018.0003

**Published:** 2018

**Authors:** Luiz Otavio Torres

**Affiliations:** 1 President Elect of the International Society of Sexual Medicine (ISSM)

**Keywords:** Prostatic Neoplasms, Hypogonadism, Prostatectomy, Therapeutics

Testosterone Replacement Therapy (TRT) was – during the last 7 to 8 decades - associated with triggering or worsening of a prostate cancer. A meta-analysis in 2005 of 19 randomized controlled trials showed no statistically significant difference in the diagnosis of prostate cancer in men using TRT or placebo (1). A pooled-analysis of 18 population-based longitudinal studies comprising 3886 men with prostate cancer (PCa) and 6438 matched controls showed no association between endogenous androgen levels and the risk of developing PCa (2).

However, more controversial is the use of TRT in men after the treatment of a prostate cancer. But, during the last years more and more studies are showing that we can use – at least in a selected group – TRT after radical prostatectomy (RP) in symptomatic hypogonadal men with no impact in the oncologic outcomes. We conducted a Medline search to find publications on TRT after RP and we summarized what we have found in literature.

Early in 2004, Kaufman and Graydon (3) reported 7 men under TRT after curative radical prostatectomy for PCa (6 men with Gleason 6 and 1 with Gleason 7). No biochemical recurrence (BCR) - as defined by consecutive increasing PSAs and patient referral for salvage radiotherapy - occurred after a follow up period of 19 months. In 1 patient, TRT was initiated in the early postoperative period due to hot flashes and low energy. There was no pattern of time of initiating TRT after RP in that series.

In 2005, Agarwal and Oefelein (4) treated 10 hypogonadal symptomatic men after RP - Gleason 6 in 2 patients, Gleason 7 in 7 patients and Gleason 8 in 1 patient - also with no biochemical recurrence after a mean follow-up of 19 months. All patients initiated TRT at least after one year of RP.

In 2008, Nabulsi et al. (5) reported 22 men under TRT after PCa – 13 with Gleason 6, 7 with Gleason 7 and 2 with Gleason 8. One patient had BCR (Gleason 8) after 12 months on TRT. Mean follow up was 24 months. Mean time after RP to initiate TRT was 11 months (range 2.5 to 118).

Also in 2008, Davila et al. (6) reported a series of 14 men after RP (mean Gleason score 6.2) receiving testosterone with a mean follow-up period of 12 months with no biochemical recurrence. No mention on the time after RP when TRT was initiated.

In 2009, Khera et al. (7) presented a study in which 57 symptomatic hypogonadal men were submitted to TRT after RP. The mean Gleason score was 6.57. TRT was initiated with an average of 36 months (range 1 to 136 months) after RP. In a mean follow up of 13 months (range 1 to 99 months) there were no BCRs.

In 2010, Isbarn et al. (8) reported the results of a questionnaire sent to urologists asking about TRT after RP. 69 patients were suitable and all of them had organ-confined prostate cancer. Median follow up was 19 months (range 6-72). Median time from RP to TRT was 24 months. There were no BCRs.

Also in 2010, Sathyamoorthy et al. (9) presented a series of 133 men on TRT after RP. In a mean follow-up of 363 days, there were no biochemical recurrences even in the high risk group (8 patients with Gleason greater than or equal to 8, positive margins or node positive disease).

In 2012, Matsushita et al. (10) reported 71 men who initiated TRT after RP with a median Gleason of 7. Men received TRT after a median time of 18 months (range 6-34) of surgery. In a median follow up of 19 months (range 9 to 35) BCR occurred in just one patient, 33 months after RP and 15 months after starting the TRT.

In 2013, Pastuzak et al. (11) reviewed 103 hypogonadal patients under TRT after RP and 49 non-hypogonadal men after RP (reference non treated group). In the treatment group, there were 77 men with low/intermediate risk cancer and 26 with high risk cancer (Gleason 8 or greater, positive surgical margins or positive lymph nodes). In the reference group, there were 34 men with low/intermediate and 15 with high risk cancer. The median interval between RP and TRT was 12.3 months. Median follow up was 27.5 months (range 6.2 to 189.3). Biochemical recurrence occurred in 4 men in the treatment group (3.9%) and in 8 patients in the reference group (16.3%), demonstrating that - in this particular group of patients - BCR incidence was higher in the non-treated group compared to the treated group. All patients with BCR – in both groups – had high risk cancer.

In 2016, Ory et al. (12) reported a series of 82 men with treated or untreated prostate cancer submitted to TRT, 22 after radical prostatectomy (14 with low/intermediate and 8 with high risk cancer). The mean follow up was 41 months (22-57). There is no information on how long after RP the TRT was initiated. There were no biochemical recurrences among these 22 men.

**Table:** All these data are summarized in the table below.

Author	Number	Time to initiate TRT after RP	Follow-up	Biochemical recurrence
Kaufman and Graydon (3)	7	Variable	Variable	0
Agarwal and Oefelein et al. (4)	10	> 1 year	Mean 19 months	0
Nabulsi et al. (5)	22	2.5-118 months	24 months	1 (Gleason 8)
Davila et al. (6)	12	Not informed	12 months	0
Khera et al. (7)	57	3 months	1-99 months	0
Isbarn et al. (8)	69	24 months	6-72 months	0
Sathyamoorthy et al. (9)	133	Not informed	Mean 363 days	0
Matsushita et al. (10)	71	6-34 months	9-35 months	1
Pastuszak et al. (11)	103	Mean 12.3 months	Mean 27.5 months	4*
Ory et al. (12)	22	Not informed	22-57 months	0
**Total**	**506**			**6 (1.19%)**

* 4 men in the TRT group (3.9%) and 8 in the non-treated group (16.3%).

**The analysis of all these data raises two questions:**

1. Do we have good evidence to conclude that testosterone hormone therapy after radical prostatectomy is safe ?
2. Is there any evidence that it is safe to initiate TRT one year after radical prostatectomy?

These data are very reassuring that we can use TRT in selected men submitted to RP, since biochemical recurrence of prostate cancer is very low: only 1.19%! In the study with a control untreated group, the incidence of BCR was higher than in the treated group (11). It is also important to highlight that among the 6 patients who experienced biochemical recurrence, 5 were in the high risk cancer group.

The data also tell us that the time to initiate TRT after RP is very unpredictable among studies. We have series initiating TRT after 2.5, 3, 6, 12 and 24 months after TRT. But in the study that showed the higher incidence of biochemical recurrence, TRT was initiated 12.3 months after surgery!

Some groups of experts and also some scientific societies have issued opinions on the subject:

Buvat et al. (13) stated: – “Men successfully treated for prostate cancer and suffering from confirmed, very symptomatic Testosterone Deficiency may be candidates for TRT after a prudent interval (depending on the type of cancer treatment), if there is no evidence of residual disease, and if they had an initial tumor contained in the prostate gland, which was not with high grade Gleason score. This must be a very individual decision”.

Khera et al. (14) affirmed the same statement two years later during the Fourth International Consultation for Sexual Medicine (ICSM 2015) promoted by the International Society of Sexual Medicine (ISSM): “Men successfully treated for prostate cancer with confirmed symptomatic TD are candidates for TRT, after a prudent interval (depending on type of cancer treatment), if there is no evidence of residual cancer”.

The European Association of Urology (EAU) guidelines on Male Hypogonadism (15) says: “Offer TRT cautiously in symptomatic hypogonadal men who have been surgically treated for localized prostate cancer and who are currently without evidence of active disease (...): treatment should be restricted to those patients with a low risk for recurrent prostate cancer (... ) and should not be started before one year of follow-up”.

Kaplan et al. (16) said that “the studies have not found a higher than expected risk of prostate-cancer progression or recurrence in men that received TRT that were previously treated for prostate cancer. PSA should be undetectable following radical prostatectomy over a 6 months period”.

There is a current Clinical Trial, randomized placebo controlled, FDA approved for TRT in hypogonadal men starting three months after radical prostatectomy: NCT00848497 (http://clinicaltrials.gov/ct2/show/NCT00848497).

In conclusion: “Do we have enough evidences to safely treat a man with hypogonadism one year after radical prostatectomy for prostate cancer ?” YES !!

This revision reassures us that selected hypogonadal symptomatic men after curative treatment for prostate cancer with radical prostatectomy can safely receive testosterone replacement therapy, since biochemical recurrence rate of the tumor is very low.

“Do we have enough evidences to safely treat a man with hypogonadism one year after radical prostatectomy for prostate cancer ? YES !

The data above showed us that it is also safe to offer TRT earlier – just three months after surgery ! And the inclusion criteria for the FDA approved Clinical Trial on TRT after RP is three months after surgery with two undetectable PSAs. So why one year after treatment would not be safe ?

But we are at the moment – despite with very reassuring data – with still a small number of patients submitted to radical prostatectomy under TRT: longer follow-up studies and larger sample sizes are needed to stablish the real safety of TRT after RP and to also standardize the time after radical prostatectomy we should initiate TRT.

But just a final personal input: if we consider that administration of testosterone to a symptomatic hypogonadal man with a history of treated prostate cancer to turn him into an eugonadal man would increase the risk of prostate cancer recurrence, why don’t we turn all our eugonadal post radical prostatectomy patients into an hypogonadal status in order to prevent an eventual prostate cancer progression ?
